# Supplementary figures and images for: Bacterial infection promotes tumorigenesis of colorectal cancer via regulating CDC42 acetylation
Source: PLoS Pathog. 2023 Feb 22;19(2):e1011189. doi: 10.1371/journal.ppat.1011189 (PMC9987831; doi:10.1371/journal.ppat.1011189)

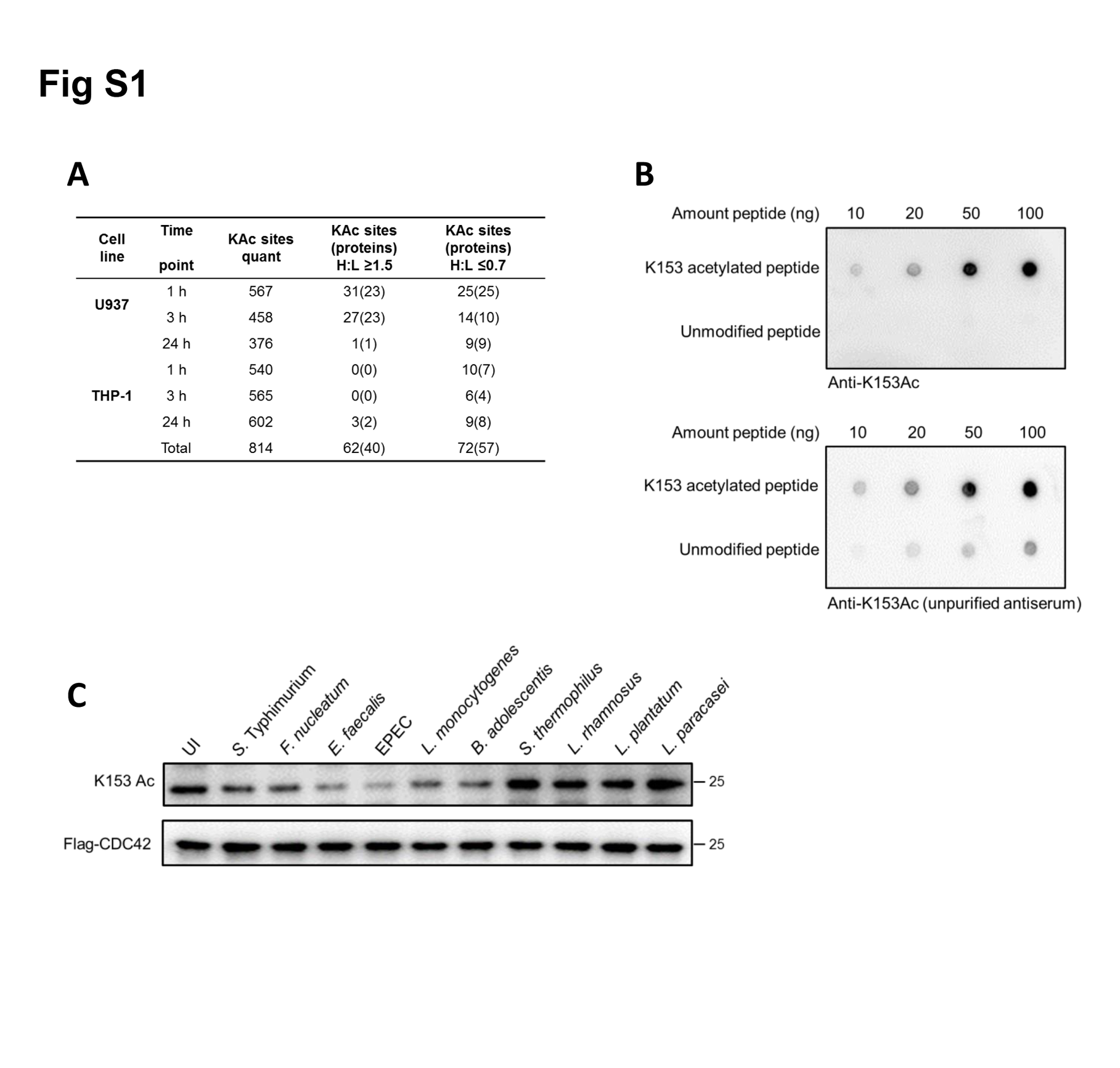

Supplement: S1 Fig — (A) Increased and decreased H:L plots for acetylated peptides in THP-1 and U937 cells are shown. (B) Characterization of CDC42 specific acetyl-K153 antibody. Specificity of CDC42 K153 acetyl-antibody was determined by dot blot assay. A nitrocellulose membrane was spotted with different amounts of acetyl-K153 peptide or unmodified peptide, and detected with purified K153 Ac antibody or unpurified antiserum, respectively. (C) K153 acetylation and several pathogenic bacteria and gut-friendly bacteria. HEK293T cells overexpressing CDC42 were infected by five pathogenic bacteria (S. Typhimurium, F. nucleatum, Enterococcus faecalis, Enteropathogenic Escherichia coli (EPEC) and L. monocytogenes) or gut-friendly bacteria (Bifidobacterium adolescentis, S. thermophiles, L. rhamnosus, L. plantarum and L. paracasei) at MOI of 100 individually, and the cells were harvested for further IP and WB experiments after 1 h. K153 acetylation was measured by IP with anti-Flag antibody and followed by WB with anti-K153Ac. (TIF) [file ppat.1011189.s001.tif]

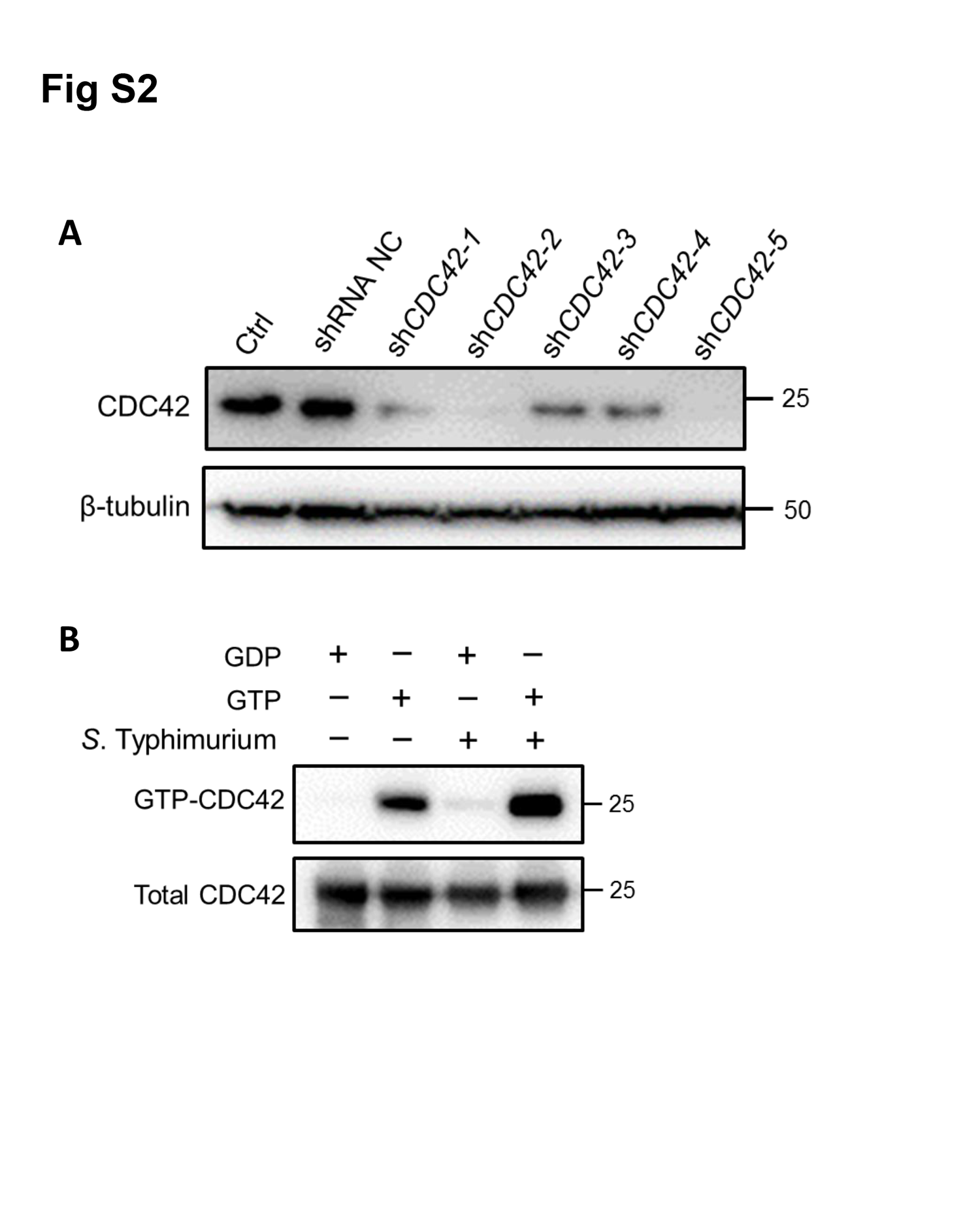

Supplement: S2 Fig — (A) HEK293T-shCDC42 and HEK293T-shRNA vector control (shRNA NC) stable cell lines were established. CDC42 was knocked down in HEK293T cells by using several shRNAs. The protein expression levels were detected by WB with anti-CDC42 antibodies. (B) Activation of CDC42 by Salmonella infection. GTP-bound form of Flag-tagged CDC42 was transfected in HEK293T cells followed by Salmonella infection, and the expression levels of GTP-CDC42 were compared using PAK1-PBD IP and WB. (TIF) [file ppat.1011189.s002.tif]

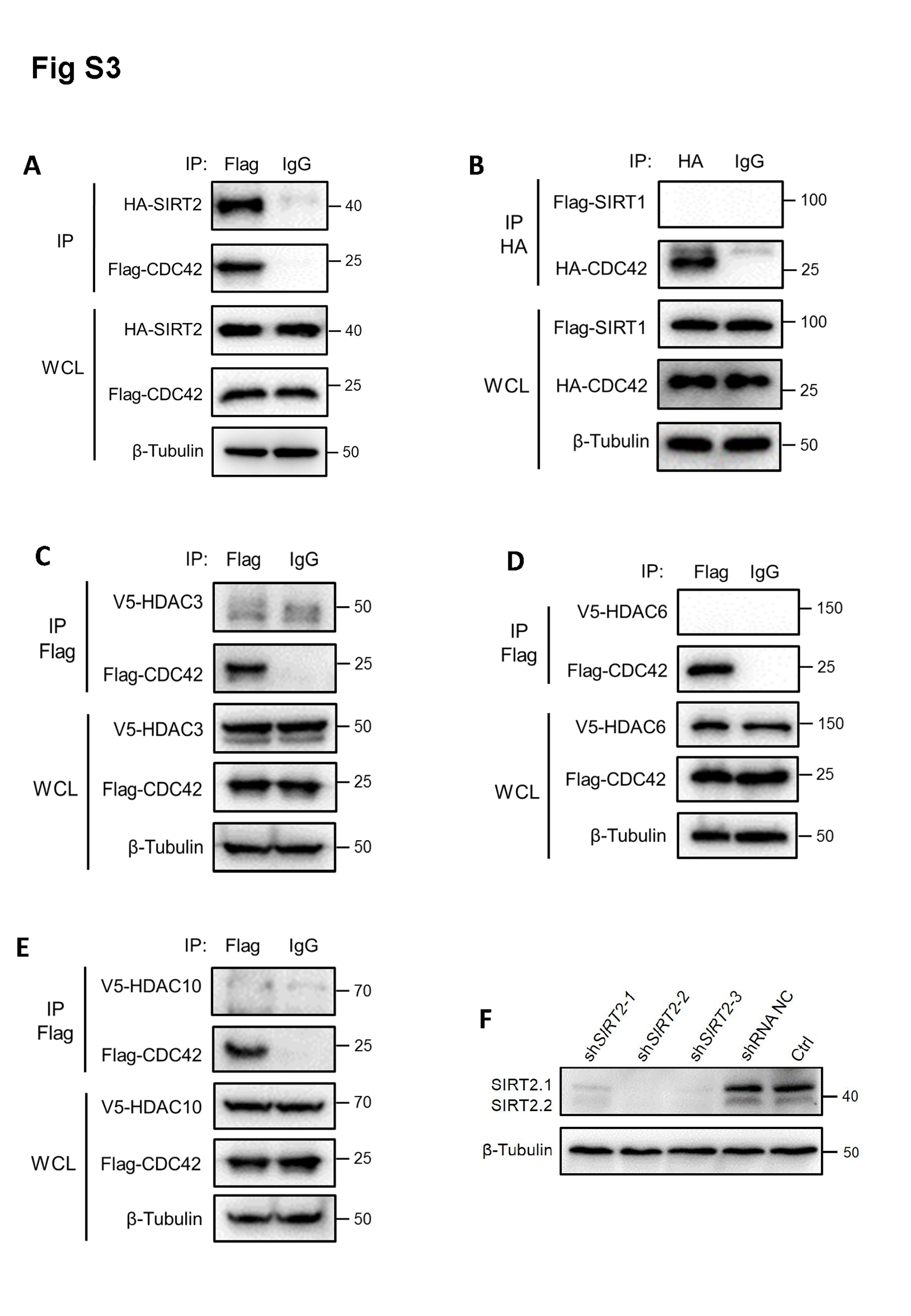

Supplement: S3 Fig — HA-SIRT2 (A), HA-SIRT1 (B), V5-HDAC3 (C), V5-HDAC6 (D), or V5-HDAC10 (E) was individually co-transfected with Flag-CDC42 into HEK293T cells. Cell lysates were used for IP with anti-Flag antibody or normal mouse IgG (as a negative control) and then analyzed by WB with the indicated antibodies. (F) SIRT2 was knocked-down in HEK293T cells by using several shRNAs. The protein levels were detected by WB with anti-SIRT2 antibodies. (TIF) [file ppat.1011189.s003.tif]

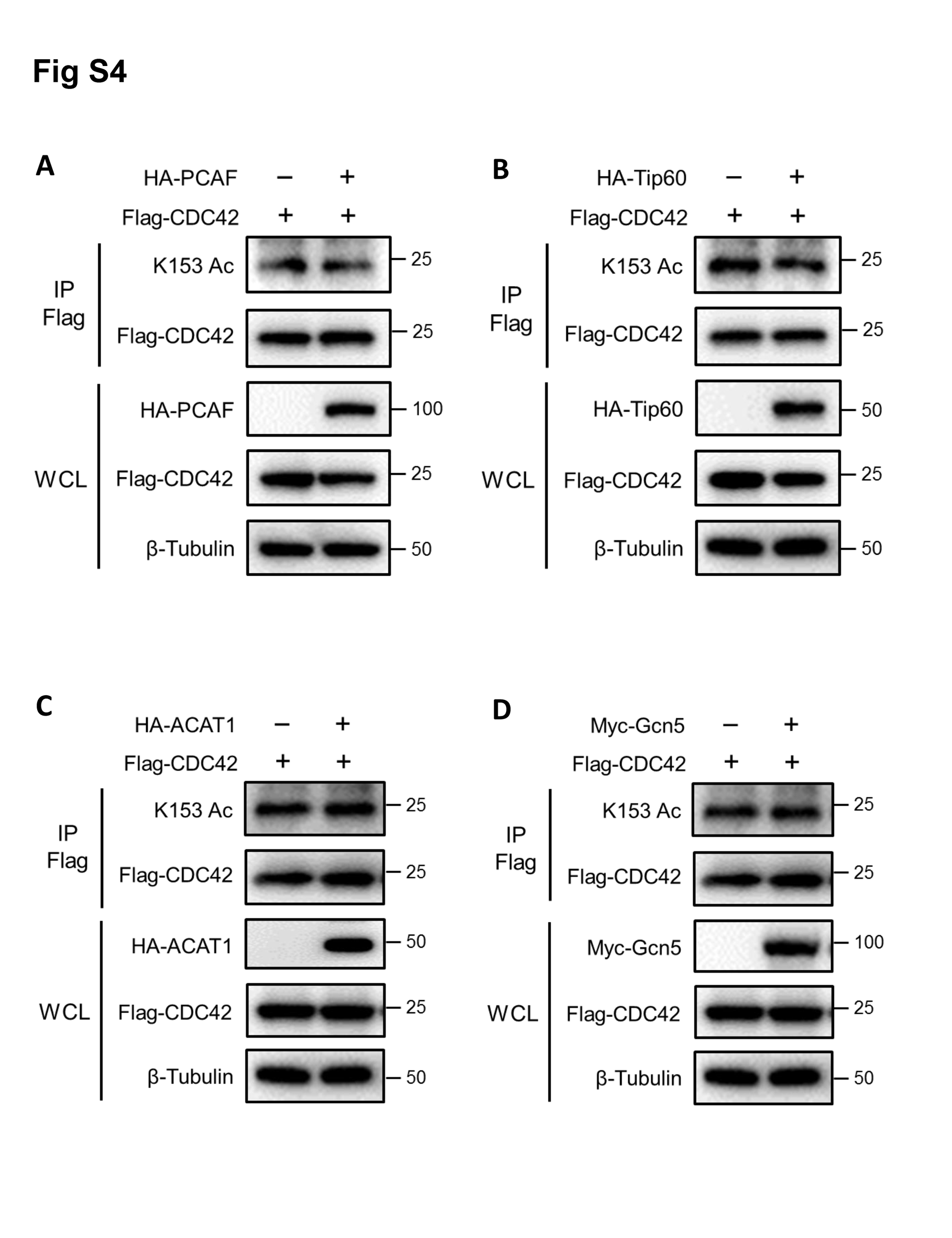

Supplement: S4 Fig — CDC42 K153 acetylation and PCAF, Tip60, ACAT1 and Gcn5. HA tagged PCAF (A), Tip60 (B), ACAT1 (C), or Myc-tagged Gcn5 was individually co-transfected with Flag-CDC42 into HEK293T cells. K153 acetylation of was determined by IP with anti-Flag antibody, while normal mouse IgG was used as a negative control, followed by WB with the indicated antibodies. (TIF) [file ppat.1011189.s004.tif]

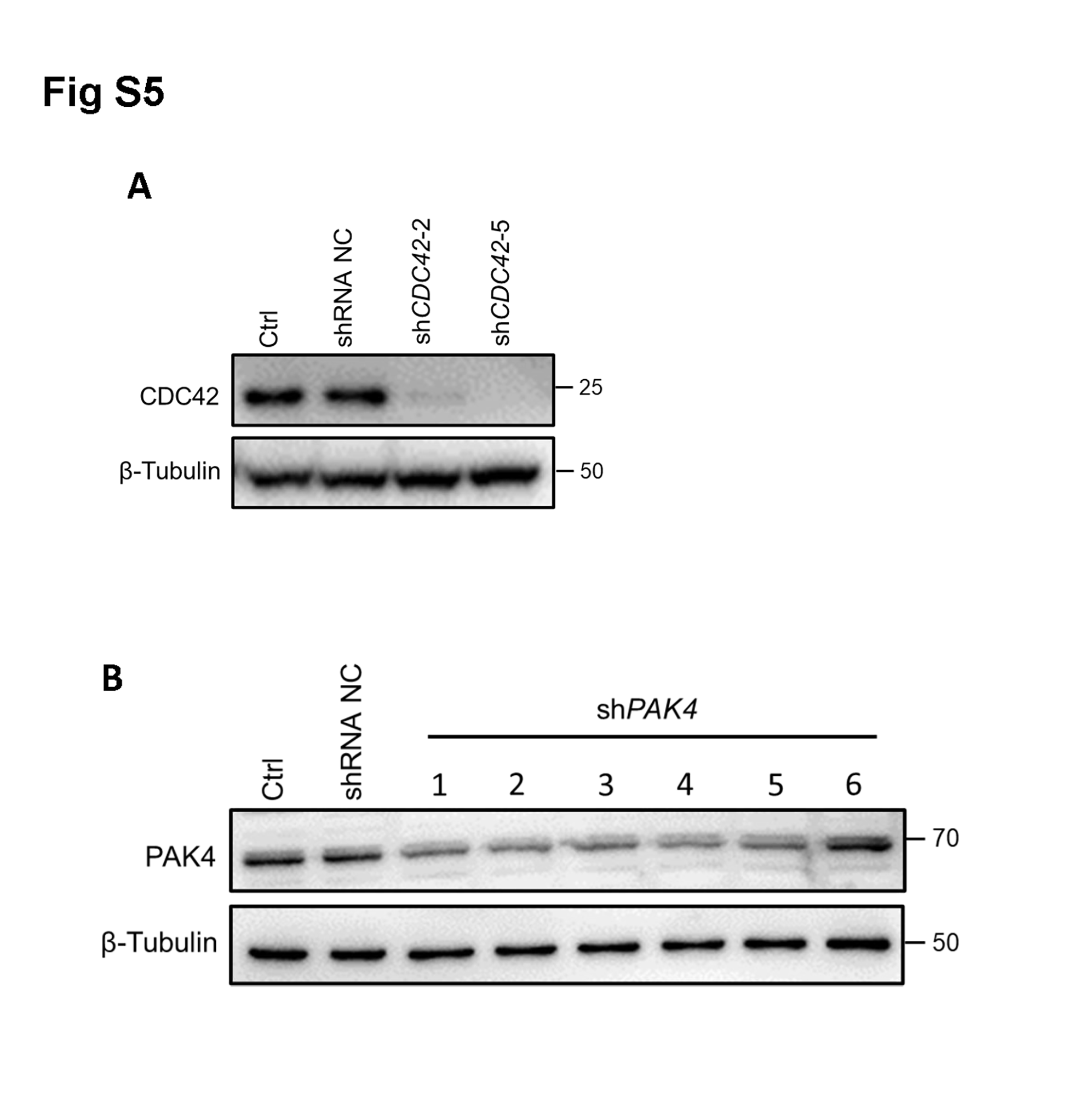

Supplement: S5 Fig — (A) HEK293T-shCDC42 and HEK293T-shRNA NC stable cell lines were established. CDC42 was knocked down by two shRNAs in HCT116 cells. (B) HEK293T-shPAK4 and HEK293T-shRNA NC stable cell lines were established. CDC42 in HCT116 cells was knocked-down by several shRNAs. (TIF) [file ppat.1011189.s005.tif]

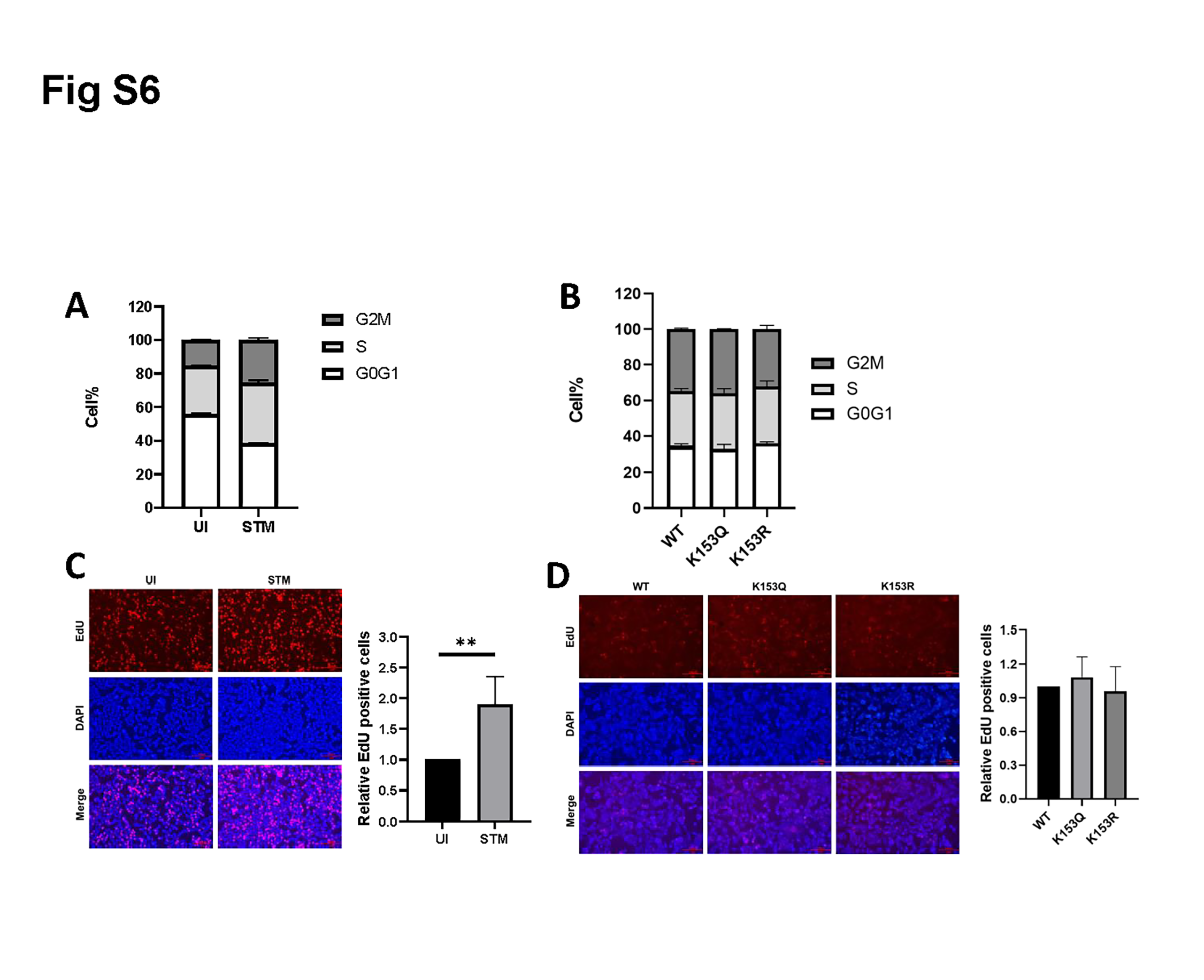

Supplement: S6 Fig — (A) Percentage of HCT116 cells in the different stages of the cell cycle after stimulation with (STM) or without (UI) S. Typhimurium. Cell cycle stage distribution was determined by flow cytometry analysis after PI staining. Representative cell cycle profiles for 24 h.p.i. are shown. (B) Percentage of HCT116 cells stably expressing CDC42-WT, CDC42 K153Q, or CDC42 K153R in the different stages of the cell cycle. EdU proliferation assay of the effect on S. Typhimurium-stimulated HCT116 cells (C) and CDC42 WT or QR mutants of CDC42 K153 in HCT116 cells (D). **p<0.01. (TIF) [file ppat.1011189.s006.tif]

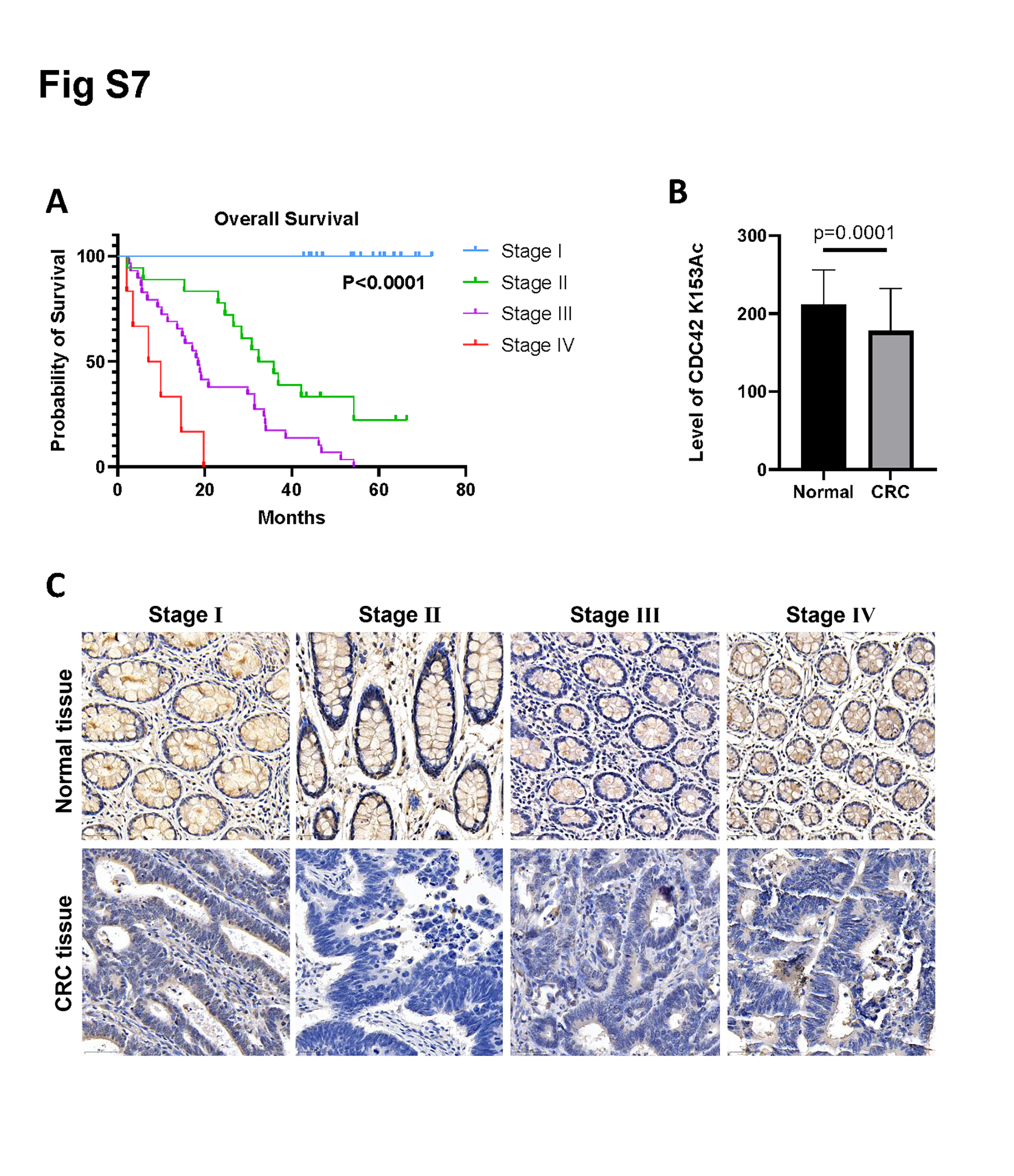

Supplement: S7 Fig — (A) Kaplan-Meier analysis of overall survival rates of CRC patients at different stages of CRC. (B) CDC42 K153 acetylation level was significantly lower in colorectal adenocarcinoma tissues than in adjacent normal colorectal tissues as determined by IHC. The average value of IHC intensity ± SD were quantitated by modified H-score from two groups of 69 patient samples is presented. (C) CDC42 K153 acetylation level of 69 human CRC specimens with different CRC stages was detected by IHC. (TIFF) [file ppat.1011189.s007.tiff]
