# Supplementary material for: Bacterial infection promotes tumorigenesis of colorectal cancer via regulating CDC42 acetylation
Source: PLoS Pathog. 2023 Feb 22;19(2):e1011189. doi: 10.1371/journal.ppat.1011189 (PMC9987831; doi:10.1371/journal.ppat.1011189)
Supplement: S2 Table — (DOCX) [file ppat.1011189.s009.docx]

**Supplementary Table 2**

| **Full Hairpin Sequence of GIPZ Lentiviral shRNAmir** | | |
| --- | --- | --- |
| sh*CDC42*-1 | TGCTGTTGACAGTGAGCGATTGGTAAAACATGTCTCCTGATAGTGAAGCCACAGATGTATCAGGAGACATGTTTTACCAACTGCCTACTGCCTCGGA | |
| sh*CDC42*-2 | TGCTGTTGACAGTGAGCGAGGCGATGGTGCTGTTGGTAAATAGTGAAGCCACAGATGTATTTACCAACAGCACCATCGCCCTGCCTACTGCCTCGGA | |
| sh*CDC42*-3 | TGCTGTTGACAGTGAGCGAATGGTGCTGTTGGTAAAACATTAGTGAAGCCACAGATGTAATGTTTTACCAACAGCACCATCTGCCTACTGCCTCGGA | |
| sh*CDC42*-4 | TGCTGTTGACAGTGAGCGATGGGCGATGGTGCTGTTGGTATAGTGAAGCCACAGATGTATACCAACAGCACCATCGCCCACTGCCTACTGCCTCGGA | |
| sh*CDC42*-5 | TGCTGTTGACAGTGAGCGATAAAAGCAATGTTTAAATCAATAGTGAAGCCACAGATGTATTGATTTAAACATTGCTTTTAGTGCCTACTGCCTCGGA | |
| sh*SIRT2*-1 | TGCTGTTGACAGTGAGCGACTGGACGAGCTGACCTTGGAATAGTGAAGCCACAGATGTATTCCAAGGTCAGCTCGTCCAGCTGCCTACTGCCTCGGA | |
| sh*SIRT2*-2 | TGCTGTTGACAGTGAGCGACAGCGCGTTTCTTCTCCTGTATAGTGAAGCCACAGATGTATACAGGAGAAGAAACGCGCTGGTGCCTACTGCCTCGGA | |
| sh*SIRT2*-3 | TGCTGTTGACAGTGAGCGCGGACTTCCTGCGGAACTTATTTAGTGAAGCCACAGATGTAAATAAGTTCCGCAGGAAGTCCATGCCTACTGCCTCGGA | |
| sh*PAK4*-1 | TGCTGTTGACAGTGAGCGACCGACTGAAGAACCTGCACAATAGTGAAGCCACAGATGTATTGTGCAGGTTCTTCAGTCGGGTGCCTACTGCCTCGGA | |
| sh*PAK4*-2 | TGCTGTTGACAGTGAGCGACAAGAAGATGGACCTGCGCAATAGTGAAGCCACAGATGTATTGCGCAGGTCCATCTTCTTGATGCCTACTGCCTCGGA | |
| sh*PAK4*-3 | TGCTGTTGACAGTGAGCGATCGATCATGAATGTCCGAAGATAGTGAAGCCACAGATGTATCTTCGGACATTCATGATCGACTGCCTACTGCCTCGGA | |
| sh*PAK4*-4 | TGCTGTTGACAGTGAGCGACAGGGTGAAGCTGTCAGACTTTAGTGAAGCCACAGATGTAAAGTCTGACAGCTTCACCCTGCTGCCTACTGCCTCGGA | |
| sh*PAK4*-5 | TGCTGTTGACAGTGAGCGCGAGGCGCGAGCTGCTCTTCAATAGTGAAGCCACAGATGTATTGAAGAGCAGCTCGCGCCTCTTGCCTACTGCCTCGGA | |
| sh*PAK4*-6 | TGCTGTTGACAGTGAGCGACCTCAAAGCCATGAAGATGATTAGTGAAGCCACAGATGTAATCATCTTCATGGCTTTGAGGGTGCCTACTGCCTCGGA | |
| **Primers for real-time RT-PCR** | | |
| Name | Forward primer | Reverse primer |
| *mmp-2* | GCAGGGCGGCGGTCAC | CGAAGGCAGTGGAGAGGAAGG |
| *mmp-9* | GACGCCGCTCACCTTCACTC | GGAACCACGACGCCCTTGC |
| *e-cadherin* | AAAGGCCCATTTCCTAAAAACCT | TGCGTTCTCTATCCAGAGGCT |
| *gapdh* | TATAGCCCCATCGTGTAGTCAGAAC | ACGCCTGCTTCACCACCTT |
| *Salmonella*-specific primers | TATAGCCCCATCGTGTAGTCAGAAC | TGCGGCTGGATCACCTCCTT |
| 18S rRNA | AGGGGAGAGCGGGTAAGAGA | GGACAGGACTAGGCGGAACA |
