# Supplementary material for: Bacterial infection promotes tumorigenesis of colorectal cancer via regulating CDC42 acetylation
Source: PLoS Pathog. 2023 Feb 22;19(2):e1011189. doi: 10.1371/journal.ppat.1011189 (PMC9987831; doi:10.1371/journal.ppat.1011189)
Supplement: S3 Table — (DOCX) [file ppat.1011189.s010.docx]

|  | **17 CRC patients** | | | | **CRC tissue microarray** | | | |
| --- | --- | --- | --- | --- | --- | --- | --- | --- |
|  |  |  | **CDC42-K153 acetylation in tumor tissues** | |  |  | **CDC42-K153 acetylation in tumor tissues** | |
| **Characteristics** | **Value** | **%** | **Lower than adjacent tissues** | **Higher than adjacent tissues** | **Value** | **%** | **Lower than adjacent tissues** | **Higher than adjacent tissues** |
| Age median (range),yrs | 65 (43-81) |  |  |  | 58 (27-82) |  |  |  |
| Sex |  |  |  |  |  |  |  |  |
| Male | 9 | 53% | 7 | 2 | 35 | 51% | 28 | 7 |
| Female | 8 | 47% | 8 | 0 | 34 | 49% | 26 | 8 |
| TNM stage |  |  |  |  |  |  |  |  |
| I | - |  |  |  | 18 |  |  |  |
| II | 11 |  | 9 | 2 | 19 |  |  |  |
| III | 6 |  | 6 | 0 | 26 |  |  |  |
| IV | - |  |  |  | 6 |  |  |  |
| Survival time (range), months | - |  |  |  | 31.5 (72.2-2.1) |  | 29.9 (72.2-2.1) | 51.3 (66.4-3) |
| Alived | - |  |  |  | 22 |  | 16 | 6 |
| Dead | - |  |  |  | 47 |  | 38 | 9 |

**Supplementary Table 3. Clinical characteristics of the colorectal cancer (CRC) parients.**
