# Supplementary material for: Bacterial infection promotes tumorigenesis of colorectal cancer via regulating CDC42 acetylation
Source: PLoS Pathog. 2023 Feb 22;19(2):e1011189. doi: 10.1371/journal.ppat.1011189 (PMC9987831; doi:10.1371/journal.ppat.1011189)
Supplement: Attachment — Submitted filename: Responses.docx [file ppat.1011189.s011.docx]

Note: The comments of reviewers are in **BLACK**, which are followed by our responses in **BLUE**.

**Point-by-point Responses to Reviewer's comments**

**Reviewer #1:**

In the manuscript titled “Bacterial infection promotes tumorigenesis of colorectal cancer via regulating 2 CDC42 acetylation”, Wang et al. investigated how S. Tm infection altered the lysine acetylome and found that among 72 KAc sites, acetylation of K153 of CDC42 was reduced. The authors provided evidence showing that reduced acetylation levels of CDC42 K153 can be observed in HEK293T cells in response to diverse pathogens. They subsequently demonstrated that S. Tm infection led to reduced binding between CDC42 and PAK4 and suggested that this phenotype resulted from the decreased acetylation of CDC42 K153. Wang et al. went on and identified SIRT2 and p300/CBP as a deacetylase and acetyltransferases for CDC42, respectively. Further, the authors showed that CDC42 K153 acetylation levels correlate with the phosphorylation status of p38 and JNK, cell survival, tumor cell migration and invasion. Lastly, the authors explored the relationship between CDC42 K153 acetylation and tumorigenesis in the large intestine using a murine CRC model and patient data. Overall, the concept of this manuscript is interesting, and a significant proportion of the experimental evidence supports the authors' conclusions. However, the clinical relevance of the core concept, and the flaws in experimental design and interpretation have dampened the enthusiasm for this manuscript.

Major Issues:

1. While the overall concept is interesting, the clinical link between Salmonella and CRC is weak. Salmonella is rarely reported to be overrepresented in or contribute to CRC development.

Response: We appreciate the reviewer’s full understanding, concise summary and critical comments to this study. In fact, many studies have shown that *Fusobacterium nucleatum* is highly enriched in CRC tissues and promotes the occurrence and development of CRC and chemoresistance. Interestingly, a comprehensive analysis of the tumor microbiome showed that more bacteria were found in tumor tissues than in adjacent normal tissues, and *Salmonella* were found in CRC (Nejman *et al.*, Science 2020, Table S4). Gram-negative *Salmonella enterica* is an intracellular pathogen to both humans and animals. *Salmonella* infection poses a major public health concern worldwide. It has been reported that antibody levels against *Salmonella* flagellin were higher in CRC and pre-cancer cases than in controls, suggesting a possible link of *Salmonella* to CRC development (Katao *et al*., Nutr Cancer 2013). The increased risk of CRC was observed among patients with *Salmonella* infection (Mughini-Gras *et al.*, PLoS One 2018 and Duijster *et al.*, Epidemiol Infect 2021). In addition, *Salmonella-*infected mice have been used as a model to study the relationship between bacterial infection and CRC development (Lu *et al*., Oncogenesis 2014). *Salmonella* effector protein AvrA could increase the incidence of colorectal tumors in a mouse CRC model (Lu *et al*., Neoplasia 2016).

In this work, we examined several bacteria including *Salmonella*, *F. nucleatum*, EPEC, *Enterococcus faecalis* and *Listeria monocytogenes* and found all of them could inhibit the acetylation of CDC42 K153 (Supplementary Figure 1C). That suggests these bacteria may contribute to the development of CRC using similar mechanisms to that of *Salmonella*. *Salmonella* was used as a representative bacterium to study the relationship between bacterial infection and development of CRC in this work.

2. The evidence provided by the authors is insufficient to support their conclusion that the reduced binding between CDC42 and PAK4 results from the weakened interaction of CDC42 E171 and PAK4 R489, which the authors claimed to be caused by the reduced acetylation of K153. The author noted that CDC42 is activated during S. Tm infection and showed that GTP-bound CDC42 WT and K153 mutants have a similar affinity towards PAK1. However, no such experiments with PAK4 were performed; therefore, it is unclear whether the activation or the reduced acetylation is responsible for reduced PAK4 binding. Additionally, it is unclear whether other PTMs cause CDC42 to display reduced affinity towards PAK4 during STM infection.

Response: The crystal structure of CDC42 and PAK4 complex suggests there might exist interactions between CDC42 E171 and PAK4 R489 (Ha *et al*., PNAS 2018). Based on the CDC42 crystal structure, we speculate that K153 may interact with E171 through hydrogen bond. Decrease of CDC42 K153 acetylation will increase the positive charge of K153, and may increase K153 interaction with the negatively charged E171 residue. Enhanced binding of K153 to E171 could disrupt the interaction between CDC42 E171 and PAK4 R489.

We showed that GTP-bound CDC42 WT and K153 mutants have a similar affinity towards PAK1, and did not determine GTP-bound CDC42 affinity towards PAK4. The reason is that the CST kit (# 11859, Cell Signaling Technology) we used to detect the activity of CDC42 is based on the binding of CDC42 to PAK1. The principle is that GTP-bound CDC42 is active and can bind to intended proteins. The kit uses PAK1 as an intended protein. Therefore, the activity of CDC42 is determined by measuring the amount of CDC42 bound to the beads. In addition, we tested the effects of K153Q (acetylated form) and K153R (unacetylated form) on the activity of CDC42, and found that the activities of CDC42 WT and these two variants were comparable (Figure 2E), indicating that QR mutation did not affect the activity of CDC42. To avoid any confusion, we have revised our statement. Now it reads: “Expression level of the active GTP-bound form of CDC42 WT or K153QR mutants in HEK293T cells with endogenous CDC42 knockdown was detected by CDC42 Activation Assay Kit (CST)”. Line 885-Line 887

Moreover, we found that K153Q and K153R mutation did not significantly affect their binding to PAK1 (Figure 2D), while K153R mutation reduced the binding of CDC42 to PAK4, and K153Q enhanced the binding of CDC42 to PAK4 (Figure 2C), indicating that acetylation of K153 affected the binding of CDC42 and PAK4, instead of changing CDC42 activity.

In our proteomics experiment, trypsin digested peptide fragments were enriched with pan-acetyl-lysine antibodies, so only lysine acetylation could be identified. So far, we do not find other modifications at CDC42 K153 in the literature, but this question is very interesting and worth further studying.

3. The relevance of the murine model presented in this paper is unclear. C57BL/6 mice are Nramp-/-, which makes them susceptible to S. Tm infection. When antibiotics such as streptomycin is used, they succumb to S. Tm infection in about a week. Suppose these mice are not pre-treated with antibiotics, as described in this paper. In that case, they are resistant to S. Tm colonization even with an inoculum 40-fold higher than described in this paper (REF: PMID: 12704158). It is, therefore, unclear whether S. Tm colonized the mice at all for the duration of the experiments, and if it did, how did the mice survive 14 weeks of infection? Moreover, the authors provided no evidence related to the S. Tm population in the gut at any time point of the experiments. Lastly, it is difficult to comprehend how S. Tm can be present inside these mice's tumors without systemic spread, which Nramp-/- C57BL/6 mice are highly susceptible to.

Response: We fully appreciate the reviewer’s expertise on murine model of *Salmonella* infection. It is very fortunate for our work to be evaluated by the reviewer (I don't know who the reviewer is – but thank the editor for finding him/her!)

We did spend a lot of time on mouse model since it is quite important to this work. As the reviewer said, mice failed to survive high dose of infection in this model. Thanks to our previous experience in mouse models of *Salmonella* infection (mBio 2021; Infect Immun 2021; Emerg Microbes Infect 2019; J Infect Dis 2017; Future Microbiol 2017; PLoS Pathog 2017), we final made it. We tried 1x10^6^ CFU, 1x10^5^ CFU, 1x10^4^ CFU, 1x10^3^ CFU, 1x10^2^ CFU or even 50 CFU to successively infect C57BL/6 mice by oral gavage. We found that 50 CFU infection per mouse could ensure most mice survive the whole experiment period. After 14 weeks, we collected mice feces and used *Salmonella* specific 16S rDNA primers to determine bacterial burden. It was found that *Salmonella* did exist in the feces of mice in the infected group at the end of experiment, but not in the uninfected group (Figure 7B). We also checked *Salmonella* burden during the experiment, and the results showed that *Salmonella* was present in feces in the whole experiment period. In addition, we used *Salmonella* LPS specific antibody to detect the mice intestinal tissues at the 14th week, immunofluorescence staining showed that *Salmonella* was present in the infected mice tissues (Figure 7D). We assume that 50 CFU is too low to induce systemic infection and *Salmonella* could only colonize in the mice intestine, so that mice could survive the experiment. In the previous study on *Salmonella* and CRC from by Sun’s group, they used 1x10^6^ CFU to infect C57BL/6 mice, and *Salmonella* could still be detected 45 weeks post infection (Lu *et al*., Oncogenesis 2014).

Minor issues:

1. The authors did not cite the paper in which the structure of PAK4:CDC42 complex.

Response: We have added the reference of PAK4:CDC42 complex structure (Ha *et al*., PNAS. 2018). Line 161

2. The authors suggested that reduced binding between CDC42 and PAK4 leads to reduced PAK4 activation and, therefore, the reduced phosphorylation of JNK and p38. However, this conclusion is not well supported because they provided no evidence showing PAK4 phosphorylation supports JNK and p38 phosphorylation.

Response: Sorry for this confusion. The literature shows that the phosphorylation of PAK4 Thr474 represents its active state (Wang *et al*., Structure 2011), and binding of CDC42 to PAK4 could activate PAK4 (Baskaran *et al*., EMBO Rep. 2012 & Wang *et al*., Expert Opin Ther Pat. 2021). It has been shown that activation of PAK4 is related to the increase of phosphorylation of JNK and p38 (Ha *et al.*, J Biol Chem. 2008; Huang *et al.*, Biochem Biophys Res Commun. 2007 & Ramos-Álvarez *et al.*, Am J Physiol Gastrointest Liver Physiol. 2020). Therefore, we propose that the decrease of JNK phosphorylation and p38 phosphorylation is due to the weakened interaction between PAK4 and CDC42. Moreover, two references (Ha *et al.*, J Biol Chem. 2008 & Huang *et al.*, Biochem Biophys Res Commun. 2007) have been added. Line 241

3. The isotope-labeled experiment can benefit from a more explicit description in the main text.

Response: Thanks for the suggestion. We have added more experimental details in the revision. “We summed up eXtracted Ion Current (XIC) of all isotopic clusters associated with the identified amino acids sequence in the light label cluster and heavy partner and calculated the ratio between two heavy and light label partners. We also assessed the significance of outlier ratios using significance A calculation method. Only peptides with average 1.5 fold change of acetylation in two states and significance A values less than 0.05 were considered up or down regulated by infection”. Line 119-Line 124

“Protein concentration was determined using a BCA protein assay and 5 mg proteins in two states mixed at equal ratio”. Line 504-Line 505

4. The authors did not provide evidence why the shRNA knock down of endogenous CDC42 did not impact the expression of the mutants.

Response: We listed the sequence of CDC42 shRNA in the Supplementary Table 2. The shRNA targets the 3'UTR region rather than the gene coding region of CDC42, so it won’t impact the expression of the mutants, which are expressed by inserting (only) the protein-coding sequence into a vector. This information has been updated in the material and method section. Line 552-Line 554

5. “CDC42 K153 116 acetylation may be universally present in nature” is a strong conclusion based on the alignment of sequences from five organisms.

Response: Based on the previous five species of organisms, we analyzed eight more species and found that CDC 42 K153 is indeed highly conserved (attached with the alignment results). In addition, previous study (Knyphausen *et al*., Small GTPases. 2015) also showed that this site is highly conserved. To avoid any overstatement, we have revised the sentence. Now it reads: “CDC42 K153 acetylation may be universally present”. Line 145

**Reviewer #2:**

In this work Wang et al. characterize the role of de/acetylation of a Rho GTPase, cell division cycle 42 (CDC42), in regulating Salmonella-induced tumorigenesis. CDC42 is highly expressed in CRCs and has previously been identified as an important player in cancer development. The authors present data that the acetylation of CDC42 at lysine 153 was reduced during Salmonella infection of cancer cells, which may impair the binding of its downstream effector PAK4. CDC42 was deacetylated by SIRT2 and acetylated by p300/CBP. Non-acetylated CDC42 reduced apoptosis and promoted the migration and invasion ability of colon cancer cells by increasing the phosphorylation of p38 and JNK. In addition, they tested the correlation between bacterial-induced tumorigenesis and CDC42 K153 acetylation in mice model and in in patients with colorectal cancer. They propose a model in which Salmonella-mediated deacetylation of CDC42 regulates PAK pathway to promote the colorectal tumorigenesis. This work established a novel connection in bacterial infection, host PTM regulation, and clinical phenotypes.

Major Issues:

1. In general, the introduction is not well organized, which makes the significance of the study unclear. Figure legends and methods are not clearly described. For example, how does GTP-CDC42 detect? How is CDC42 acetylation peptide calculated?

Response: Sorry about this confusion in the introduction section. We have reorganized the introduction section. Now it reads: “*Salmonella* is one of the most common foodborne pathogens in the world, which can cause a variety of diseases in different hosts, from mild enterocolitis to systemic fatal infection . Recent research indicates that there are more bacteria in tumor tissues than in normal tissues, and *Salmonella* has been found in tumor tissues of colorectal cancer (CRC), breast cancer, ovarian cancer, and even lung cancer”. Line 57-Line 61

We used the CST kit (#11859, Cell Signaling Technology) to detect the activity of CDC42. Since GTP-bound CDC42 is active and can bind to intended proteins, the kit utilizes the CDC42 binding to PAK1 to determine the activity of CDC42. The kit uses PAK1 as an intended protein. Therefore, the activity of CDC42 is determined by measuring the amount of CDC42 bound to the beads. Line 573-Line 577

We apologize for unclarity in Figure legend and method sections. We have checked and revised Figure legend and method sections. For example:

In Materials and Methods

**Lentivirus construction and infection**:

We have added “The shRNA targets the 3'UTR region rather than the gene coding region of genes, and the sequences of shRNA were shown in the Supplementary Table 2”. Line 552-Line 554

**CDC42 activity assay**

We have revised “CDC42 activity assay” to “CDC42-GTP activity assay”. Line 569, and we have added “Since GTP-bound CDC42 is active and can bind to intended proteins, the kit utilizes the CDC42 binding to PAK1 to determine the activity of CDC42. The kit uses PAK1 as an intended protein. Therefore, the activity of CDC42 is determined by measuring the amount of CDC42 bound to the beads. Specifically, cells were lysed in ice-cold lysis buffer with protease inhibitor cocktail (Sango) and cleared with glutathione-agarose beads”. Line 573-Line 577

In Figure legends, we have modified the legends of Figure 2E to “Expression level of the active GTP-bound form of CDC42 WT or K153QR mutants in HEK293T cells with endogenous CDC42 knockdown was detected by CDC42 Activation Assay Kit (CST)”. Line 885-Line 887”

We have added description of fold intensity next to Figures 5E (original Figure 5D), 5F (original Figure 5E), 5G (original Figure 5F), Figure 6G (original Figure 6I), Figure 6I (original Figure 6J), Figure 7F, Figure 7G, Figure 7H (original Figure 7I), and Figure 7I (original Figure 7H).

All the raw files were processed with a single MaxQuant run. We summed up eXtracted Ion Current (XIC) of all isotopic clusters associated with the identified AA sequence in the light label cluster and heavy partner and calculated the ratio between heavy and light label partners. For CDC42, the ratio H:L in U937 cell lines after infected 1 h was 0.56 which means the acetylation of K153 site was down-regulated at 1 h. We also assessed the significance of outlier ratios using significance A calculation.

2. The differences between some western blot bands are hardly distinguished (eg. Figures 5D, 5F, 6I, 6J, 7F, 7G, 7I) while the authors declare there are significant decrease or increase. Attach histograms next to them will help the readers to understand the data more easily.

Response: Thanks for the suggestion. We have added histograms of fold intensity next to Figures 5E (original Figure 5D), 5F (original Figure 5E), 5G (original Figure 5F), Figure 6G (original Figure 6I), Figure 6I (original Figure 6J), Figure 7F, Figure 7G, Figure 7H (original Figure 7I), and Figure 7I (original Figure 7H).

3. Due to the lack of proper positive controls in some assays, some sub-figures should be put together to claim the conclusions, such as Fig3B and FigS3A-3D, Fig4A-B and FigS4A-3D, Fig5B and FigS5B.

Response: Very good suggestion. We have reorganized figures accordingly. For example, Figure 3B was moved to supplemental as Supplementary Figure 3A, Supplementary Figure 5B was moved to main text as Figure 5C. However, we think that Figure 4A and 4B are important for acetyltransferase screening, so they were not combined with the other data in Supplementary Figure 4.

Minor issues:

1. In Figure 1, the authors did not explain that why lysine-acetylome was conducted. Is the total acetylation pattern altered in host cells during Salmonella infection? This could be detected by immunoblotting analyses with specific anti-KAc antibody. Also, they did not explain clearly why selected CDC42 for further investigation among more than 50 candidates.

Response: So far, many papers have shown that pathogen infection could alter host protein post translational modification patterns including phosphorylation, ubiquitination, glycosylation, *etc* (Stévenin *et al.*, Cell Chem Biol. 2022; Dawson *et al*., PLoS Pathog. 2018). For example, *Salmonella* infection leads to extensive ubiquitination of various effectors and several outer membrane proteins in host (Fiskin *et al*., Mol Cell. 2016). Our group has been working on protein acetylation in *Salmonella* for many years (Ren *at al*., Trends Microbiol. 2017), so it is quite natural for us to explore whether *Salmonella* infection could change host protein acetylation pattern. Therefore, we used SILAC to determine macrophage protein acetylation dynamics during *Salmonella* infection at whole genome scale. In fact, we observed some differences of total acetylation pattern between the infected and uninfected groups by western blot with pan-acetyl-lysine antibody, and finally chose 1 h, 3 h and 24 h post infection for further SILAC analysis. As we expected, SILAC data showed that many host proteins involved in infection had acetylation dynamics. Notably, we found that CDC42 and its interaction proteins (Figure 1C) were acetylated upon *Salmonella* infection. Since CDC42 is crucial for many key cellular signal pathways and plays an important role in infection (Ireton *et al*., Cell Microbiol. 2014; Quintero *et al*., Small GTPases. 2015; Fiorentini *et al*., Cell Death Differ. 2003), we decided to determine the role of acetylation of CDC42 in *Salmonella* infection. All the candidates identified from SILAC have been evaluated, and some of them will be further studied.

2. In Figure 1D, the shown MS/MS spectrums of modified peptides is incomplete. The coverage of amino acids is not enough. Besides, how acetylation modification rate is calculated? The authors could refer to related documents (PMID: 30323948, 32504010).

Response: Thanks for the suggestion. Figure 1D is a representative mass spectrum analysis of the peptide containing CDC42 K153Ac. The spectrum covering the region from 100 to 1200 m/z which includes the peptide containing the acetylated lysine 153. The MS/MS spectrum was generated by using the pLabel software, and it contained the essential information of mass to charge ratio, retention time, and peptide sequence. The reference (PMID: 32504010) has been cited in the revision. Since we used pan-acetyl-lysine antibodies to enrich acetylated peptides in our proteomics experiment, and unacetylated peptides can not be harvested for mass spectrum analysis, so it is hard to calculate the coverage of CDC42 amino acids. Regard to the peptide of AVK (*) YVECSALTQK in the supplementary table1-U937 1 h, the score at K153 acetylation of CDC 42 is 68.83 (greater than 20 is considered to be reliable). For more detailed information about this peptide, you can refer to the Supplementary Table 1-U937 1 h (Line 49 CDC42). We summed up eXtracted Ion Current (XIC) of all isotopic clusters associated with the identified amino acids sequence in the light label cluster and heavy partner and calculated the ratio between two heavy and light label partners. Then the ratio was normalized, the median of the total ratio population was shifted to 1 and data was analyzed using the MaxQuant software version 1.3.0.5.

3. In Supplementary Figure 1B, the specificity of the antibody to K153-acetylated (K153Ac) CDC42 remains to be determined. To exclude the cross-link reaction with acetylation modification of the other sites within CDC42, it should be tested with a transfected K153 CDC42 mutant.

Response: Good suggestion. We used anti-K153Ac antibody to check CDC42 and its variants, and found that K153R had relative weak acetylation signal, but K153Q had similar acetylation signal to that of CDC42 (please see figure below, left panel). Furthermore, lysine 144 of CDC42 has been reported to be acetylated (Knyphausen *et al*., Small GTPases. 2015), but we did not detect the acetylation of K144 in our SILAC data. In order to further verify the specificity of anti-K153Ac antibody, we here used the site-specific incorporation of acetyl-lysine in recombinant proteins system (Neumann *et al*., Nat Chem Biol. 2008) to generate site specific acetylated CDC42 at K153 or K144 to evaluate the specificity of anti-K153Ac antibody. Western blot result showed that purified CDC42 K153Ac had a strong signal, but CDC42 K144Ac did not have such signal (please see figure below, right panel). Therefore, the above results, together with Supplementary Figure 1B showed that the anti-K153Ac antibody used in this study is specific.

4. In Supplementary Figure 1C, the infection efficiency of different bacteria should be determined.

Response: HEK293 cell line is an epithelial cell line from primary human embryonic kidney. We used several pathogens (*i.e*., *S.* Typhimurium, *F. nucleatum*, *E. faecalis*, EPEC, and *L monocytogenes*) and probiotics (*i.e.*, *S. thermophiles*, *L. rhamnosus*, *L. plantarum*, and *L. paracasei*) to treat HEK293 cells at MOI of 100. We then harvested cells for further IP and WB experiments after 1 h. So, the infection efficiencies of these bacteria should be comparable. Accordingly, the figure legend of Figure 1C has been updated.

5. In Figure 2A，why CDC42 acetylation specifically affect CDC42 binding with PAK4 other than PAK1. Is there a conserved site in PAK1 and PAK4 in the protein structure?

Response: We compared the protein sequences of PAK1 and PAK4 and found that R489 is conserved in PAK1 and PAK4 (please see figure below). The crystal structure of CDC42 and PAK4 complex showed that PAK4 R489 interacts with CDC42 E171 (Ha *et al*., PNAS. 2018). However, the crystal structure of PAK1 and CDC42 complex is not available. We also found that *Salmonella* infection decreased the interaction between CDC42 and PAK1 instead of PAK4 (please see the figure in the next question). In addition, a previous study showed that *Salmonella* infection did not affect the interaction between CDC42 and PAK1 (Sun *et al*., Nat Microbiol. 2018). So, the question is very intriguing and can be answered by deciphering the structure of CDC42 and PAK1 complex.

6. In Figure 2C，it seems that S. Typhimurium infection also decreased the binding between CDC42 and PAK1 (lane 1 and lane 3).

Response: We showed the binding between CDC42 and PAKs after *Salmonella* infection in Figure 2B. Western blot showed that *Salmonella* infection decreased the interaction between CDC42 and PAK1 a little bit. But this slight decrease might not have biological significance compared to the drastic decrease of PAK4 binding. In addition, a previous study also showed that *Salmonella* stimulates pro-inflammatory signaling through CDC42 and its effector kinase PAK1 and the interaction between CDC42 and PAK1 is not affected by *Salmonella* infection (Sun *et al*., Nat Microbiol. 2018).

7. SIRT2 deacetylates CDC42 K153. However, in Figure 3G, the CDC42 acetylation ration did not increase after knock-down of SIRT2 (Lane 2-3), which is contradictive with the conclusion of Figure 2E.

Response: The expression of SIRT2 is relatively low, and bacterial infection could increase dramatically SIRT2 expression (Eskandarian *et al*., Science. 2013; Gogoi *et al*., PLoS Pathog. 2018; Bhaskar *et al*., Elife. 2020). Therefore, SIRT2 knockdown had little effect on CDC42 K153 acetylation under non-infection condition (Figure 3G, Lane 2-3), while increased significantly CDC42 K153 acetylation under *Salmonella* infection condition (Figure 3G, Lane 4-5).

8. Non-acetylated CDC42 K153 suppresses phosphorylation of p38 and JNK (Figure 5B). However, acetylation of CDC42 K153 only regulated the phosphorylation of p38 but not JNK during Salmonella infection (Figure 5C). Instead, the phosphorylation level of JNK seems decreased in WT-CDC42 transfected cells than in CDC42 K153Q and in K153R group (lane3 in Figure 5C). How to explain this?

Response: Yes, we showed in Figure 5B that non-acetylated CDC42 K153 suppressed the phosphorylation of p38 and JNK under non-infection condition. However, acetylated CDC42 K153 could suppress the phosphorylation of p38 during *Salmonella* infection (Figure 5D, original Figure 5C). WB showed transfection of CDC42 K153 mutation could increase the phosphorylation level of JNK compared with WT-CDC42. We speculate that acetylation status of both K153Q and K153R has been fixed, while WT-CDC42 still has K153 acetylation dynamics. Moreover, bacterial infection is a complex condition, and other unknown factors may be involved in regulating JNK phosphorylation. This observation suggests that JNK signaling pathway is important to bacterial infection and may have more complex regulatory signals compared with p38 signaling pathway. We have added this part to the discussion. Line 421-Line 426

9. Figure 5G-5I only indicated that non-acetylated CDC42 K153 inhibited the STS-induced apoptosis. However, there is a gap to say “Our results suggested that Salmonella could also prevent CRC cell apoptosis by decreasing CDC42 acetylation.” (Line 396).

Response: Agree. We have revised accordingly. Now it reads: “low acetylation level of CDC42 K153 could inhibit CRC cell apoptosis”. Line 416-Line 417 (original Line 396)

10. Compared with WT, the expression of mmp-2 and mmp-9 increased both in K153Q and K153R group. The author should explain this.

Response: Although the expressions of *mmp-2* and *mmp-9* were higher in K153Q and K153R, the expressions of *mmp-2* and *mmp-9* in K153R was still significantly higher than those in K153Q. Moreover, the mimicked acetylation and non-acetylation by amino acids substitution only simulate the state of acetylation to a certain extent, which may have limitations.

11. PAK1 phosphorylation level appeared to be higher in the AOM/DSS-treated mice group with Salmonella infection. However, CDC42 acetylation does not affect its binding with PAK1 (Figure 2D), nor control the phosphorylation of PAK1 (Figure 5D). The author should explain this.

Response: Our results showed that the acetylation of CDC42 has little effect on the binding of PAK1, but the regulatory mechanism in the AOM/DSS induced CRC model with *Salmonella* infection is highly complicated, so other factors might enhance phosphorylation of PAK1 besides CDC42. Line 436-Line 439

12. Line 327-329, expression and of CDC42 increased of CRC tissues. In the meanwhile, K153 acetylation level decreased. Author should discuss which factor determines the tumorigenesis.

Response: “Activation of CDC42 facilitates tumor cell survivability and microadenoma formation, so CDC42 is considered to be crucial for tumorigenesis. Moreover, CRC cells overexpressing CDC42 K153R showed a stronger migration and invasion ability, thus suggesting that the low acetylation level of CDC42 K153 may contribute to the migration and invasion of CRC cells. Therefore, both the expression level of CDC42 and the acetylation of CDC42 K153 play an important role in the development of CRC”. We have added this part to the discussion. Line 427-Line 432

13. In FigS6B, it is not clear how to define the high and low CDC42 K153 acetylation levels.

Response: Sorry about this confusion. In fact, CDC42 K153 has a certain level of acetylation under normal physiological conditions. According to immunohistochemistry and H-score (Line 637-Line 639, Line 1054), if the acetylation level of CDC42 K153 in tumor tissues is lower than that in adjacent tissues, it is defined as a low acetylation. Otherwise, it is defined as a high acetylation. We have added this information to the methods. Line 637-Line 639

14. There is no obvious evidence in the figures to match the authors’ descriptions, such as line 330-332 and line 368-371.

Response: Sorry for this confusion. We analyzed the expression of CDC42 and CDC42 K153 acetylation in all adjacent normal and cancer tissues by immunohistochemistry and quantitated data by modified H-score (PMID: 31345636). We have added this information to the figure legend, and Figure 8B has been redrawn.

15. In the discussion section, the author should also discuss the mechanism by which Salmonella induces the acetylation of CDC42 protein, such as bacterial derived LPS stimulation, iNOS pathway activation, or the direct effects of bacterial deacetylases like cobB. On the other hand, it should be considered that the effects of CDC42 K153 acetylation on Salmonella intracellular invasion and replication.

Response: Good suggestion. We also used LPS to stimulate cells, and detected the acetylation level of CDC42 K153 at 1 h after stimulation, but we did not observe significant acetylation level changes, which suggests that the acetylation level change of K153 was not due to LPS stimulation. In addition, the decrease of K153 acetylation level was observed in cells treated by most Gram-positive and Gram-negative pathogens, which indicates that decrease of K153 acetylation level may be a common phenomenon caused by bacterial exposure.

16. Line 150, CDC42 instead of CDC142; Line 281, K153 instead of K153R.

Response: Thanks. We have corrected “CDC142” to “CDC42” in Line180 (original Line150). but K153R in Line 309 (original Line 281) is correct. Our results showed that K153R-expressing cells had lower e-cadherin level compared to WT and K153Q groups.
