# Supplementary material for: Bacterial infection promotes tumorigenesis of colorectal cancer via regulating CDC42 acetylation
Source: PLoS Pathog. 2023 Feb 22;19(2):e1011189. doi: 10.1371/journal.ppat.1011189 (PMC9987831; doi:10.1371/journal.ppat.1011189)
Supplement: Attachment — Submitted filename: Response-revision2.docx [file ppat.1011189.s012.docx]

Note: The comments of reviewers are in **BLACK**, which are followed by our responses in **BLUE**.

**Point-by-point Responses to Reviewer's comments**

Reviewer #1: Minor issues:

1. While the authors provided some evidence supporting the roles of Salmonella in CRC, the overall link is still weak as compared to some other bacterial pathogens, such as Fusobacterium, which the authors noted. It will be helpful to include the information provided in the rebuttal letter in the Introduction section to better support the rationale underlying using Salmonella as the model pathogen.

Response: Thank you for the suggestion. We have revised the introduction and added the information as follows. “For example, *Fusobacterium nucleatum* is a well-known bacterium that promotes the development of CRC through TLR4 /Kap1/NRF2 signal pathway. Interestingly, several studies have shown that *Salmonella*, which causes digestive tract infection as the common symptom, may be related to the development of CRC. It has been reported that antibody levels against *Salmonella* flagellin were higher in CRC and pre-cancer cases than in controls, suggesting a possible link of *Salmonella* to CRC development. The increased risk of CRC was observed among patients with *Salmonella* infection. *Salmonella* effector protein AvrA could increase the incidence of colorectal tumors in a mouse CRC model.” Line 65- Line73

2. The authors’ reasoning for using PAK1 as a control for their claims that “reduced acetylation of CDC42 weakens its interactions with PAK4”, is appreciated. However, the authors cannot exclude the possibility that other PTMs cause CDC42 to display reduced affinity towards PAK4 during STM infection. If the authors cannot provide additional evidence, the conclusion should be toned down.

Response：We agree with the reviewer’s suggestion. This study aims to explore the role of CDC42 K153 acetylation in CRC tumorigenesis, and our data support that K153 acetylation is involved in the interaction between CDC42 and PAK4. Currently, we cannot exclude that other PTMs caused by *Salmonella* infection may also change the affinity between CDC42 to PAK4. It is an important question and worth studying in the future. The relevant contents have been added in the Discussion section. Line 407- Line 411.

3. The relevance of the mouse model still warrants questions. While the authors titrated the inoculum to optimize Salmonella infection, it is curious how S. Tm can infect B6 without antibiotic treatment, especially given the low inoculum dose (50 CFU). If the infection did lead to changes in tumorigenesis (either by incidence or pathology), logically, the infection should have led to some degree of inflammation, which was not reported in this study. In addition, it is unclear whether the mice used in this study contain Enterobacteriaceae (E. coli, Klebsiella, etc; if the mice were sourced from Jackson, they should be Enterobacteriaceae-free, but should have Enterobacteriaceae if they were sourced from Charles River or other vendors). Because the differences in 16S sequences between Salmonella and other Enterobacteriaceae members are so slight, it is unlikely to distinguish one from another using 16S qPCR. As such, if Salmonella infection leads to the bloom of endogenous Enterobacteriaceae (https://www.pnas.org/doi/10.1073/pnas.1113246109 , https://www.cell.com/cell-host-microbe/pdfExtended/S1931-3128(18)30630-9), the detection method presented in this paper cannot distinguish what was detected. In summary, in the absence of additional supporting information, the relevance of this model should be toned down.

Response：The mouse model in this study is based on the colon cancer induced by AOM/DSS. It has been reported that DSS can lead to aggravation of enteritis and high level of inflammation, which is conducive to the formation of tumors. We focus on the role of *Salmonella* infection in CRC tumorigenesis, so mice inflammatory responses were not examined. In fact, if we use antibiotic-pretreated B6 mice, even low dose bacterial challenge will kill all the mice in one week. Therefore, we established the current mouse model and try to study the role of CDC42 acetylation in CRC tumorigenesis.

These two references the reviewer mentioned here demonstrate that *Salmonella* infection leads to the bloom of endogenous enterobacteriaceae. The mice used in our study are not Enterobacteriaceae-free, so it is very likely that endogenous enterobacteria may jointly lead to the development of CRC in the presence of pathogenic bacteria such as *Salmonella*. We have discussed the possibility and cited these two references in the Discussion section. Line 447- Line 450.

The primers we used for *Salmonella* detection are *Salmonella* specific. The reverse primer targets 16S rDNA sequence, and the forward primer targets *Salmonella*-specific sequence adjacent to 16S rDNA gene. So, this pair of primers can only amplify *Salmonella* sequence. We have updated the relevant information in Table S2 and Figure legend (Line 991- Line 992).
